# PSYCHOMETRICS OF THE PHYSICAL RESILIENCE SCALE IN OLDER ADULTS LIVING WITH DEMENTIA: PROXY RESPONSES

**DOI:** 10.1093/geroni/igae098.1678

**Published:** 2024-12-31

**Authors:** Barbara Resnick

**Affiliations:** University of Maryland School of Nursing, Baltimore, Maryland, United States

## Abstract

Physical resilience is defined as the ability to recover or optimize function in the face of physical challenges. Physical resilience has been conceptualized as a trait focusing on the way individuals respond to stressors versus an outcome of the stressor(s) or physically challenging experience. Due to cognitive changes, including decreased ability to recall new information or aphasia, it is difficult for individuals living with dementia to respond to questionnaires. Consequently, a family member or identified proxy is often asked to respond. The purpose of this study was to determine if proxies can complete the Physical Resilience Scale for older adults living with dementia. This was a descriptive study using Rasch analysis and baseline data from the Function Focused Care for Acute Care Using the Evidence Integration Triangle trial. The first 240 patients living with dementia were included. There was evidence of reliability based on person and item separation index. There was no evidence of Differential Item Functioning (DIF) between genders although there was evidence of DIF by race on Item 7 (He/she accepted help from others). Validity was supported based on items fitting the model except for Item 10 (He/She found it difficult to ask for help from others). Controlling for age, gender, race and comorbidities there was a significant relationship between resilience and function (R2 =.08, F=.4.75, p=.001) and resilience and pain (R2 =.08, F=3.95, p.002). The Physical Resilience Scale is reliable and valid when completed by proxy reports. Future use should remove Item 10 due to redundancy.

